# Cognition in Primary Care Community Resource Directory for Individuals, Caregivers, and Providers

**DOI:** 10.1093/geroni/igab046.1338

**Published:** 2021-12-17

**Authors:** Monica Zigman Suchsland, Annette Fitzpatrick, Barak Gaster, Jaqueline Raetz, Judit Illes, Benjamin Olivari, Lisa McGuire, Basia Belza

**Affiliations:** 1 University of Washington, Seattle, University of Washington, Washington, United States; 2 University of Washington, Seattle, Washington, United States; 3 The Gerontological Society of America, Washington, District of Columbia, United States; 4 Centers for Disease Control and Prevention (CDC), Atlanta, Georgia, United States; 5 CDC, Atlanta, Georgia, United States

## Abstract

A KAER Model recommendation is to refer individuals diagnosed with dementia to resources that help them prepare for the future and services that provide ongoing support. The purpose of this project was to locate local quality services and develop a resource directory for persons with cognitive impairment for use by providers, staff, individuals, families, and caregivers. We worked with a Community Advisory Board and interviewed individuals and caregivers to understand what resources are useful and important to include in the resource directory. We built a web-based resource directory that allows users to query resources based on specific needs. We integrated the resource directory within the electronic health record for providers to include after visit summaries. A resource directory was deployed for community use, with goals of sustainability and longevity after this project is completed.

